# PAMK Ameliorates Non-Alcoholic Steatohepatitis and Associated Anxiety/Depression-like Behaviors Through Restoring Gut Microbiota and Metabolites in Mice

**DOI:** 10.3390/nu16223837

**Published:** 2024-11-08

**Authors:** Jianmei Yang, Wanyi Ou, Guiru Lin, Yuanfei Wang, Dongliang Chen, Ze Zeng, Zumin Chen, Xiaomin Lu, Aiping Wu, Chenli Lin, Yinji Liang

**Affiliations:** 1 School of Nursing, Jinan University, Guangzhou 510632, China; yjm6782022@163.com (J.Y.); owy15625565622@163.com (W.O.); gtr201820@163.com (G.L.); yuanfei_wang526@163.com (Y.W.); 15625562724@163.com (D.C.); 13717063481@163.com (Z.Z.); 18320014569@163.com (Z.C.); luxiaomin01030@163.com (X.L.); 17877723256@163.com (A.W.); 2 School of Medicine, Jinan University, Guangzhou 510632, China; 3 Health Science Center, Jinan University, Guangzhou 510632, China

**Keywords:** gut microbiome, serum metabolites, nonalcoholic steatohepatitis, anxiety/depression-like behaviors, atractylodes macrocephala koidz polysaccharide

## Abstract

Objectives: Long-term Western diet-induced non-alcoholic steatohepatitis (NASH) can lead to liver cirrhosis and NASH-associated hepatocellular carcinoma, which are end-stage liver diseases. Meanwhile, NASH is associated with mental burden and worsens as the disease progresses. Atractylodes Macrocephala Koidz (AMK) is one of the main ingredients of Shenling Baizhu San, and the effect of Polysaccharide from AMK ameliorates (PAMK), as an important medicinal ingredient of AMK, on NASH and associated anxiety/depression-like behaviors is still unclear. Methods: This study investigated the protective effect of PAMK on NASH and associated anxiety/depression-like behaviors through a Western diet-induced NASH mice model. Results: showed that PAMK decreased the concentrations of liver TC, TG, and serum AST and ALT, improving glucose tolerance, and reducing liver steatosis and fibrosis. Moreover, the expression of liver IL-6, IL-1β, TNF-α, IL-18 and MCP-1 could be reduced by PAMK significantly. Additionally, PAMK decreased anxiety/depression-like behaviors and expression of IL-6, IL-1β, TNF-α, and MCP-1 in the hippocampus. 16S rRNA gene sequencing revealed that PAMK diminished the Firmicutes/Bacteroidetes ratio and abundance of Faecalibaculum_rodentium, and increased the abundance of Muribaculaceae. This might be related to gene abundance of Pentose, the glucuronate interconversions pathway and carbohydrate enzymes (GH1, GH4). Serum metabolomics suggested that PC (18:5e/2:0), PC (16:2e/2:0), Lysopc 20:4, PC (16:0/2:0), and LPC 19:0 upregulated significantly after PAMK intervention, together with the enrichment of carbon metabolism and Citrate cycle pathways specially. Conclusions: PAMK as a potential prebiotic ameliorated NASH and associated anxiety/depression-like behaviors in mice, probably by regulating Faecalibaculum_rodentium, carbohydrate enzymes and lipid metabolites.

## 1. Introduction

With the alterations of diet habits and lifestyles, the incidence of diet-induced metabolic diseases continues to increase worldwide. As one of the fastest-growing metabolic diseases, nonalcoholic steatohepatitis (NASH), featuring lobular inflammation, steatosis and ballooning of liver tissue, is the progressive form of the NAFLD spectrum [1,2]. The existing data predict that over 40% of patients with NASH will progress to fibrosis, and the prevalence of NASH and related mortality will double by 2030 [1,2,3]. The onset of NASH always goes through four stages including liver lipid accumulation known as NAFL, fatty infiltration and liver inflammation, fibrosis, and liver cirrhosis [4]. Without intervention, this hepatocellular damage would promote collagen deposition, and ultimately evolve to hepatocellular carcinoma [5]. The occurrence of NASH is related to Western diet pattern-induced liver lobular inflammation, oxidative stress, and endoplasmic reticulum stress [6,7]. Coincidentally, evidence also implicates that Western diet-induced chronic low-grade systemic inflammation and neuroinflammation can lead to common mental disorders by the gut-brain axis in mice [8,9]. Changes in gut microbiota can have harmful and beneficial effects on health [10]. Previous research showed that the Western diet is associated with the disturbance of gut microbiota structure and diversity, and the surge of chronic inflammation, which may influence the liver and brain through the gut-liver and gut-brain axis [11,12]. Given the high prevalence and poor prognosis of NASH, it is of great clinical importance to find prevention and therapy that not only exert protective effects on the liver, but also reduce related mental disorders.

Atractylodes Macrocephala Koidz (AMK), belonging to the Asteraceae family, is a food homologous medicine with few adverse reactions, which also is the chief component in Shenling Baizhu San (SLBZS). In Traditional Chinese Medicine, AMK can be used to prevent gastrointestinal disorders, cancer, obesity, etc. [13]. The rhizome of AMK has been reported as possessing many biological activities such as anti-tumor, anti-inflammatory, anti-oxidative, anti-aging, and neuroprotective activities, and it contains sesquiterpenoids, triterpenoids, polyacetylenes, coumarins, phenylpropanoids, flavonoids, and flavonoid glycosides, steroids, benzoquinones, and polysaccharides [14]. Polysaccharides are the main active ingredients of AMK [15,16,17]. Our preliminary research found that SLBZS produced an effective improvement on NAFLD [18]. Fortunately, recent studies have also reported that polysaccharides as a prebiotic have a significant protective effect on NASH and depression [19,20,21]. For example, Astragalus polysaccharides can ameliorate hepatic inflammation and lipid accumulation by modulating the structure of gut microbiota and reducing the Firmicutes/Bacteroidetes ratio [22,23]. Similarly, large-leaf yellow tea polysaccharides can suppress the progression of NAFLD by adjusting bile salt hydrolase-related microbial genera [24]. Furthermore, poria cocos polysaccharides prevent the development of NASH by reshaping the composition of gut microbiota and mitigating inflammatory symptoms [25]. Marine algal polysaccharides also present an obvious potential for ameliorating NASH through inhibiting TGF-β1 and NF-κB levels, and downregulating the PI3K/AKT pathway [26]. Hence, polysaccharides may have a potentially protective effect on NASH and related mental disorders. Taking the above into consideration, it is still unclear how polysaccharides act through gut microbiota and metabolites, so this study used multi-omics to investigate the effect of PAMK on the liver and hippocampus through a Western diet-induced non-alcoholic steatohepatitis (NASH) mice model.

## 2. Materials and Methods

### 2.1. Extraction and Chemical Analysis of PAMK

All Atractylodes and macrocephala Koidz polysaccharides without endotoxin (purity ≥ 98%) were purchased from Shanxi Ciyuan Biotechnology Co., Ltd. (CY190105, Xi’an, China). For detecting monosaccharide components, we utilized the Thermo ICS 5000+ Ion chromatography system (ICS 5000+, Thermo Fisher Scientific, Waltham, MA, USA), accompanied with Dionex™ CarboPac™ PA20(Thermo Fisher Scientific, Waltham, MA, USA) (150 × 3.0 mm, 10 μm) liquid chromatography columns and electrochemical detector (Thermo Fisher Scientific, Waltham, MA, USA) [27]. In addition, the infrared spectrum of PAMK was obtained using a Nicolet iZ-10 Fourier transform infrared (FT-IR) spectrometer (Nicolet iZ-10, Thermo, USA) [28]. We observed that 3278.5 cm^−1^ presented the characteristic O-H stretching vibration absorption peak, and the absorption peak at 2929.3 cm^−1^ was attributed to the C-H stretching vibration. Moreover, an absorption peak at 1016.7 cm^−1^ belonged to the stretching vibration of C-O, which is a characteristic region of the glycosidic bond. PAMK is mainly composed of glucose (74.82%), mannose (16.08%), and arabinose (9.09%), which displayed that glucose was the predominant monosaccharide [29].

### 2.2. Animals and Experimental Design

**Figure 1:**
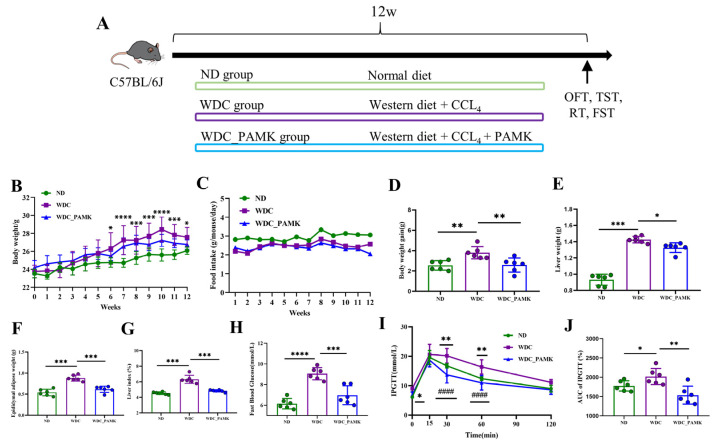
Effect of prebiotics-like PAMK on adiposity and blood glucose in NASH mice. (A) Animal experimental schedule; OFT, open field test; TST, tail suspension test; RT, rotarod test; FST, forced swimming test. (**B**) Weekly body weight of mice. * Indicates a significant alteration in body weight of ND and WDC by two-way ANOVA with Dunnett’s post hoc test. (**C**) Average daily food intake of each mouse. (**D**) Body weight gain. (**E**) Liver weight of mice. (**F**) Epididymal adipose weight of mice. (**G**) Liver index, liver weight to body weight ratio. (**H**) Fast blood glucose. *p*-value was obtained by one-way ANOVA with Dunnett’s post hoc test. (**I**) Intraperitoneal glucose tolerance test (IPGTT). (**J**) AUC, the area under the curve of IPGTT. Data are presented as the mean ± SD (*n* = 6), * *p* < 0.05 or ** *p* < 0.01 or *** *p* < 0.001 or **** *p* < 0.0001 for ND vs. WDC group; #### *p* < 0.0001 for WDC vs. WDC_PAMK group; *p*-value obtained by two-way ANOVA with Dunnett’s post hoc test.

Male C57BL/6J mice (6–8 weeks) were purchased from the Laboratory Animal Technology Co., Ltd. of Zhejiang Wei Tong Li Hua (Beijing, China). All mice were housed at a temperature of 23 ± 2 °C and humidity of 55% ± 5 under a 12 h light-dark cycle with free access to food and water at the Animal Center of Jinan University [30]. The experiment was conducted following the Ethics Committee of Jinan University, China (No. IACUC-20220301-17). Mice were randomly divided into three groups: ND (normal diet, no CCl_4_ injection, *n* = 6), WDC (Western Diet, trace CCl_4_ injection, *n* = 6), and WDC_PAMK (PAMK, Western Diet, trace CCl_4_ injection, *n* = 6) [31]. Mice in the ND group were fed a normal chow diet (ND, 5C02, Lab diet). Mice in the WDC and WDC_PAMK groups were fed a Western diet containing 17.3% protein, 48.5% Carbohydrate, 1.25% cholesterol, and 21.2% fat by weight (TP.120528A, Trophic Animal Feed High-tech Co., Ltd., Nantong, China) with daily drinking water and a high sugar solution with 23.1 g/L d-fructose (F108334, aladdin) and 18.9 g/L d-glucose (G116306, aladdin). Then, 0.2 uL/g CCl_4_ (10006464, Sinopharm Chemical Reagent Co., Ltd., Shanghai, China) of body weight was dissolved in corn oil (10%CCL_4_/corn oil) and then was injected intraperitoneally into mice in the WDC and WDC_PAMK group once a week. As a control, an equal amount of corn oil was injected into mice in the ND group once a week. The WDC_PAMK group was oral gavage with PAMK 700 mg/kg every day for 12 weeks, while the ND group and WDC group were given an equal volume of distilled water (Figure 1A). In order to facilitate understanding, we regarded the WDC group as the NASH group, and the WDC_PAMK group as the PAMK group. The body weight and food intake of the mice were recorded once a week. All mice were humanely euthanized at the 12th week, and liver tissue, blood samples, feces, brain, and hippocampus were collected [31].

### 2.3. Behavioral Tests

#### 2.3.1. Open Field Test (OFT)

The open field was conducted in four uniformly sized cells (50 cm × 50 cm × 50 cm) in a square black box and four mice were allowed to perform at the same time. The mice were brought to the testing room for adaptation for 30 min. Once beginning, four mice were placed in the center of each cell simultaneously and allowed to explore freely, recorded by camera for 5 min. The time spent in the center zone and the total distance of each mouse were recorded and counted by Etho Vision 7.0 software (Noldus, Wageningen, The Netherlands) [30]. Mice tended to cling to the walls because of thigmotaxis. When mice moved around more in the periphery, they were more pronounced with anxiety-like behaviors. Spending more time in the center of the open field indicated mice with less anxiety.

#### 2.3.2. Tail Suspension Test (TST)

The mouse’s tail was suspended on a handing rod (30 cm from the bottom of the apparatus) using tape (1–2 cm from the tip of the tail) and hook for 5 min. Three mice could be recorded and tested simultaneously, separated by black partitions. The immobility time of each mouse was recorded and counted by Etho Vision 7.0 software (Noldus, Wageningen, The Netherlands). Reduction in the time spent immobile was considered an antidepressant activity [32]. Stopping struggling, curling up, and becoming apathetic could be considered similar to depression. Experimenters recorded how long the mice stayed immobile, and quantified the extent of depression-like state.

#### 2.3.3. Forced Swimming Test (FST)

The FST was performed in three clear glass cylinders filled with water (temperature, 23–25 °C); cylinder dimensions were: height, 30 cm; diameter, 20 cm; and water level, 15 cm. Three mice were gently placed into a water tank at the same time and their behavior was recorded by video for 5 min. Movement states were analyzed by Etho Vision 7.0 (Noldus, The Netherlands). After the experiment, mice were picked up by their tails, dried gently with towels, and kept warm at room temperature. They were considered to be immobile whenever their heads floated above the surface of the water and stopped swimming [32]. Longer periods of immobility indicated more severe depression.

#### 2.3.4. Rotarod Test (RT)

The fatigue level was analyzed in a rotarod treadmill for mice (longer bio, Shanghai, China). Before the formal experiment, mice underwent adaptive training for at least 5 min. After resting for 30 min, mice were placed on the rotarod device for ten seconds recording began after their gait became stable and balanced. The rotor speed was accelerated from 0 to 4 rpm within 10 s, and from 4 rpm to 40 rpm within 300 s until mice fell. The time on the rod was counted as “latency to fall”. Shorter time on the rod meant the mice were more likely to fatigue [33].

### 2.4. Histological Analysis

After the execution of the mice, liver and brain tissues were fixed in 4% paraformaldehyde until histopathological trial, and then embedded in paraffin, sectioned, and stained with hematoxylin and eosin (H&E) or with Sirius Red for assessing fibrosis according to standard procedure. NAFLD activity score (NAS) and fibrosis were evaluated by two pathologists according to the Non-alcoholic steatohepatitis clinical research network (NASH-CRN) scoring system containing steatosis, lobular inflammation, and hepatocellular ballooning (score 0~2: non-NASH; 3~4: borderline NASH; 5~8: NASH), and the slides were blind scored [34]. The frozen liver tissue used for Oil Red O (ORO) staining was embedded in an optimal cutting temperature compound, and was sectioned to 5 μm thickness. Images were taken under a microscope (Leica, Wetzlar, Germany). Semi-quantitative analysis of the Oil Red O staining area and the Sirius Red staining fibrotic area were analyzed by image J (1.50k version) [35].

### 2.5. Biochemical Indicators Detection

After blood was collected from the mouse orbit, the blood was placed for two hours and then centrifuged at 1200 r for 10 min at 4 °C to collect serum. The serum of mice was used to detect alanine aminotransferase (ALT) and aspartate aminotransferase (AST), and liver tissue was used to detect triglyceride (TG) and total cholesterol (TC) levels with commercial assay kits (Nanjing Jiancheng Bioengineering Institute, Nanjing, China) according to protocols. An intraperitoneal glucose tolerance test (IPGTT) was performed after 12 h of fasting but free drinking. Glucose (2 mg/g body weight) in normal water was given to the mice by intraperitoneal injection. The blood glucose (BG) level was measured from tail blood at 0, 15, 30, 60, and 120 min after glucose administration (Contour TS, Bayer, Barmen, Germany) [36]. The materials used in the aforementioned experiment are listed in Table S2.

### 2.6. Real-Time Reverse Transcriptase Polymerase Chain Reaction Analysis

For determining the expression of IL-1β, TNF-α, IL-6, MCP-1, and IL-18 mRNA in the liver and IL-1β, TNF-α, IL-6, and MCP-1 in the hippocampus, 1 ug of total RNA was reverse transcribed using a complementary DNA conversion kit (R223-02, VAZYME, San Diego, CA, USA). Gene expression levels were determined by quantitative PCR (Q712, VAZYME, USA) on the CFX Connect Real-Time PCR Detection System (BIO-RAD, Hercules, CA, USA) [30,31,32,33,34,35,36]. The qRT-PCR protocol’s first step was 95 °C for 30 s, then 40 cycles for 3 s at 95 °C, 10 s at 60 °C, 15 s at 95 °C, 60 s at 60 °C, and 15 s at 95 °C. The relative expression of target genes was normalized by GAPDH expression as an internal control. The primers used in the experiment are listed in Tables S1 and S2.

### 2.7. 16 S rRNA Gene Sequencing and Analysis

Feces (>1 g) were freshly collected from the mice before behavioral tests. Once collected, they were immediately put in liquid nitrogen with 1.5 mL tubes and stored in a refrigerator at −80 °C. After extracting genomic DNA from the fecal samples, the V3-V4 hypervariable region of the 16S rRNA gene was amplified and purified (primer sequence: 5′-3′, 341F: CCTACGGGNGGCWGCAG, 806R: GGACTACHVGGGTATCTAAT). The second round of amplification products was purified using AMPure XP Beads and quantified using the ABI StepOnePlus Real-Time PCR System (Life Technologies, Carlsbad, CA, USA). Then the purified samples were sequenced and analyzed based on the PE250 mode pooling test of Novaseq 6000 (Illumina, San Diego, CA, USA). Sample quality testing and sequencing were provided by GENEDENOVO Biotechnology Company (Guangzhou, China) [37]. After the sequencing data was downloaded, we first performed quality control on the data, clustered it, removed chimeras, and obtained OTU representative sequences and abundance information. Species annotation was performed based on OTU sequences, and species abundance information at each level was obtained based on OTU abundance information statistics. Then, bioinformatics analysis such as species composition, indicator species, α diversity, β diversity, functional prediction, and environmental factor association was carried out.

### 2.8. Serum Untargeted Metabolomics

The blood was collected from the eyeballs of mice at 12 weeks and serum was obtained by centrifugation at 4 °C after stewing for 2 h, and stored at −80 °C until use. After abstracting the sample, QS samples were used for quality control. UHPLC-MS/MS analysis was performed using a Vanquish UHPLC system (Thermo Fisher, Erlangen, Germany) coupled with an Orbitrap Q Exactive™ HF-X mass spectrometer (Thermo Fisher, Germany) in GENEDENOVO Biotechnology Company (Guangzhou, China). Chromatographic separation was performed on a Hypesil Gold column (100 × 2.1 mm, 1.9 μm). The raw data files generated by UHPLC-MS/MS were processed using Compound Discoverer 3.1 (CD3.1, Thermo Fisher). Then, peaks of each metabolite were matched with the mzCloud (https://www.mzcloud.org/ (accessed on 3 November 2024)), mz Vaultand Mass Listdatabase to obtain accurate qualitative and relative quantitative results [38].

### 2.9. Statistical Analysis

To determine the association among gut microbiota, serum metabolites, biochemical parameters and behavior indicators in three groups, we performed a Spearman correlation analysis. When logarithmic transformation fails to standardize the distribution of the variable, the Wilcoxon rank sum test was used for the paired test, while the Kruskal–Wallis rank sum test, and one-way or two-way ANOVA following Dunnett’s post hoc test (GraphPad Prism 8.0, San Diego, CA, USA) were conducted to assess the differences among the three groups. Data were expressed as the mean ± standard deviation (SD). A *p*-value < 0.05 was deemed to be statistically significant. The sample size could not be calculated because the effect size and inter-individual variability were unknown, and this study was considered exploratory.

## 3. Results

### 3.1. Prebiotics like PAMK Ameliorated Adipose Accumulation and Glucose Tolerance in NASH

At first, to explore the effect of PAMK on NASH mice, we induced the NASH model through a Western diet plus microinjection of CCl_4_ (WDC). According to the results of weekly body weight and food intake (Figure 1B,C), compared to the ND group, the body weight in the NASH group significantly increased from the sixth week, and there was a downward trend in the group administrated with PAMK. However. a significant change in food intake was not observed between the NASH and PAMK groups. To reflect the states of adipose accumulation in mice, we measured several body fat indicators. Results displayed that although body weight gain (NASH vs. PAMK, 3.8 ± 0.61 vs. 2.6 ± 0.69), liver weight (NASH vs. PAMK, 1.4 ± 0.04 vs. 1.3 ± 0.06), epididymal adipose weight (NASH vs. PAMK, 0.9 ± 0.07 vs. 0.6 ± 0.07), and liver weight-to-body weight ratio increased significantly after being fed a Western diet, which were obviously ameliorated by PAMK (Figure 1D–G). Moreover, to evaluate the metabolic capacity of glucose in mice, we detected the fast blood glucose at 12 weeks and found that PAMK could effectively reduce fast blood glucose (NASH vs. PAMK, 9.1 ± 0.59 vs. 7.0 ± 0.92) in NASH mice (Figure 1H). Further, IPGTT showed that after 15 min of glucose intraperitoneal injection, glucose levels rose gradually and remained elevated significantly at 30 min and 60 min in the NASH group in contrast to the ND and PAMK groups (Figure 1I,J). It was seen that the glucose tolerance of mice decreased under the Western diet pattern, which was improved after the intervention of PAMK. Accordingly, gavage prebiotics like PAMK reduced epididymal adipose accumulation and body weight gain, and ameliorated glucose tolerance in NASH mice.

### 3.2. Prebiotics like PAMK Improved Liver Parameters and Proinflammatory Factors in NASH Mice

**Figure 2:**
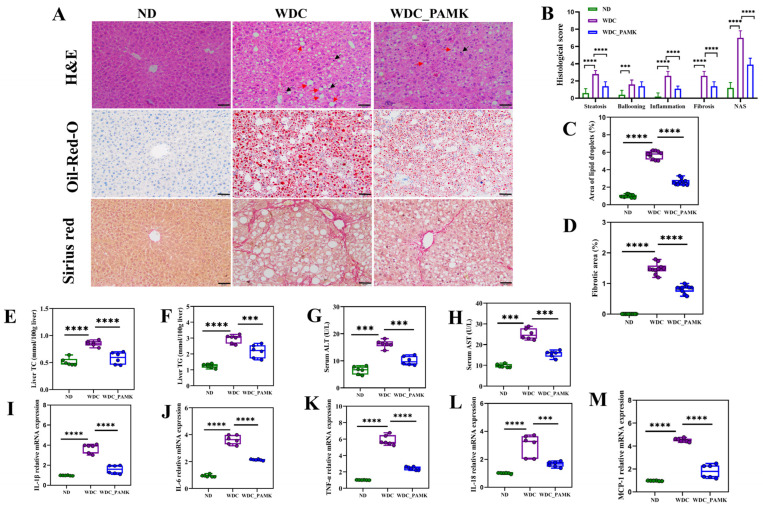
Prebiotics like PAMK administration ameliorated liver injury, lipid accumulation, inflammation, and fibrosis in NASH mice. (**A**) Representative of liver tissues stained with H&E, Oil Red O, and Sirius Red in ND, WDC, WDC, PAMK groups (original magnification ×200, scale bar: 50 μm). Red arrows point to steatosis, and black arrows point to inflammation. (**B**) NAFLD activity score including lobular inflammation score, steatosis score, hepatocyte ballooning score, and fibrosis score under ten views of liver H&E and Sirius Red slices. (**C**) Semi-quantitative analysis of lipid droplets under ten views of Oil Red O staining area. (**D**) Quantitation of Sirius Red-positive liver area. (**E**) Liver total cholesterol, TC (*n* = 6). (**F**) Liver triglycerides, TG (*n* = 6). (**G**) Concentration of serum alanine aminotransferase, ALT (*n* = 6). (**H**) Concentration of serum aspartate transaminase, AST (*n* = 6). (**I**–**M**) Relative mRNA expression of proinflammatory factors IL-1β, IL-6, TNF-α, IL-18, MCP-1 in liver. Results are presented as the mean ± SD (*n* = 6), *** *p* < 0.001 or **** *p* < 0.0001; *p*-value obtained by one-way ANOVA with Dunnett’s post hoc test.

To observe whether PAMK changes the liver pathology of NASH mice, the H&E, Oil Red O, and Sirius Red staining were performed (Figure 2A). Firstly, we observed mice in the NASH group showed abnormal liver lobular inflammation, steatosis, hepatocyte ballooning, and high density as well as big-size lipid droplets based on the H&E and Oil Red O staining liver sections, as evidenced by the risen NAFLD activity related score (Figure 2B). To further confirm our finding, liver steatosis and fibrosis were quantified. We found that the large percentage of Oil Red O staining positive area in NASH mice (Figure 2C), and high levels of liver total cholesterol and triglyceride (Figure 2E,F), were significantly alleviated by PAMK (*p* < 0.05). Subsequently, the results of Sirius Red staining revealed that PAMK effectively reduced collagen deposition with stage 2.2 (±0.4) fibrosis accompanied by a significant reduction in the fibrotic area in NASH mice (Figure 2D). Serum concentrations of ALT and AST in NASH mice increased approximately two-fold in contrast to ND mice, which were mitigated in the PAMK group (Figure 2G,H). Furthermore, to evaluate whether the mRNA expression of proinflammatory factors in the liver has changed, we used RT-PCR technology for this detection. The results displayed that there was a notable reduction of mRNA expression of liver TNF-α, IL-1β, IL-6, MCP-1, and IL-18 in the PAMK group (Figure 2I–M). To summarize, prebiotics like PAMK inhibited the development of NASH by reducing liver lipid accumulation, the mRNA expression of liver proinflammatory factors, and serum ALT and AST.

### 3.3. Prebiotics like PAMK Suppressed NASH Related Anxiety/Depression-like Behaviors

**Figure 3:**
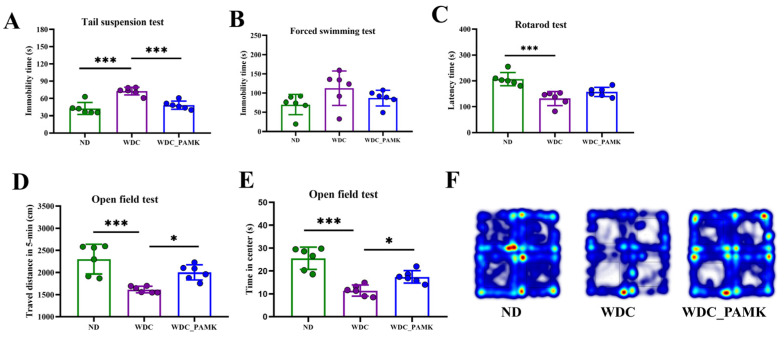
Anxiety- and depression- like behaviors were alleviated by prebiotics-like PAMK in NASH mice. (**A**) The time mice stay immobile state within 5 min in tail suspension test (TST). (**B**) The time that mice stay in an immobile state and their heads floating above surface in forced swimming test (FST). (**C**)The time mice stay on the spinning stick in rotarod test (RT). (**D**) Time spent in center area in 5 min during open field test (OFT). (**E**) Travel total distance of mice in 5 min during open field test. (**F**) The heat map of mice movement trajectory of open field test. Results are presented as the mean ± SD (*n* = 6), * *p* < 0.05 or *** *p* < 0.001; *p*-value obtained by one-way ANOVA with Dunnett’s post hoc test.

Evidence showed that obese mice induced by a high-fat and high-cholesterol diet exhibited related anxiety/depression-like behaviors [30]. Thus, we conducted a series of behavioral tests to confirm whether mice in the NASH group would exhibit anxiety/depression-like behaviors and the effects of PAMK on related neurological behaviors. We found an approximately 2-fold increase in immobility time in the NASH group compared to the ND and PAMK group during the tail suspension test (TST, *p* < 0.05, one-way ANOVA and Dunnett’s post hoc test) (Figure 3A). Similar results were observed in the forced swimming test (FST), though the immobility time of NASH mice is not significant as compared with ND mice, there was an improving trend that could be observed in the PAMK group (Figure 3B). This immobile, non-struggling, non-climbing of mice is similar to the depressive state of humans, and results of TST and FST indicated that PAMK might have a beneficial effect on depression-like behaviors in NASH mice. Additionally, a systematic review of patient-reported outcomes in NASH patients shows that they experience co-occurring symptoms of fatigue and depression in daily life, and fatigue is a powerful predictor of depression [39]. Therefore, to examine the fatigue level, we conducted rotarod in mice. The result indicated that NASH mice spent less time on rotarod in contrast to the PAMK group (Figure 3C), which meant mice in group NASH were more prone to fatigue. Moreover, the time mice stay in the center area and the total distance away from the center of the open field test are effective indicators to evaluate the anxiety and depression levels. The subsequent results of Figure 3D showed that the total travel distance in 5 min of mice in the NASH group was significantly shortened compared to the PAMK and ND group (*p* < 0.05, one-way ANOVA and Dunnett’s post hoc test). In addition, we observed that mice in the NASH group stayed in the center area for approximately half as long as in the ND group, and an obvious improvement was observed in the PAMK group (*p* < 0.05, one-way ANOVA and Dunnett’s post hoc test) (Figure 3E). The heat map of mice movement trajectory also indicated that NASH mice tended to stay around open fields in contrast to mice in ND and PAMK groups (Figure 3F). Consequently, the aforementioned results indicated that NASH mice emerged anxiety/depression-like behaviors, which could be ameliorated by prebiotics like PAMK.

### 3.4. The Neuron Injury and Inflammation of Hippocampus Were Modified by Prebiotics like PAMK in NASH Mice

**Figure 4:**
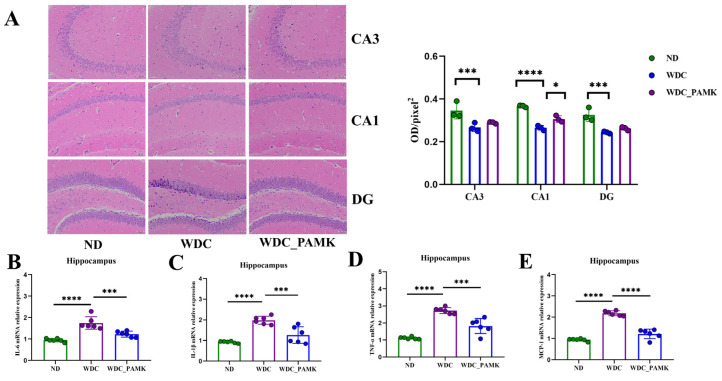
Neuronal damage and neuroinflammation in the hippocampus were ameliorated after PAMK intervention. (**A**) The representative of hippocampus stained with H&E, scale bar: 50 μm, the magnification was 200×; CA: cornu ammonis, DG: dentate gyrus; OD: Optical Density. *p*-value was obtained by two-way ANOVA with Tukey’s post hoc test. (**B**–**E**) The levels of mRNA expression of proinflammatory factors IL-6, IL-1β, TNF-α, MCP-1 in hippocampus of ND, WDC, and PAMK groups when compared with WDC group by one-way ANOVA with Dunnett’s post hoc test. Results are presented as the mean ± SD (*n* = 6), * *p* < 0.05 or *** *p* < 0.001 or **** *p* < 0.0001.

Animal models suggest that depression can impair hippocampal neurogenesis, which directly impacts stress regulation, and depression and anxiety symptoms [40]. Therefore, we used H&E slices of the hippocampus to investigate whether the hippocampal neurons of NASH mice would change, and what effects PAMK would exert. As is known, the hippocampus can be divided into the dentate gyrus (DG) and the cornu ammonis region (CA) which can be further subdivided into the CA1, CA2, and CA3 areas. Compared with the ND and PAMK groups, H&E slices of the NASH group exhibited unclear neuronal nucleoli, condensation and slight swelling, along with cell morphology and unclear structure (Figure 4A). Semi-quantitative analysis of neurons in three subdivisions of the hippocampus revealed that a reduction in the density of normal neuron cells in the hippocampus (23% for CA1, 28% for CA3, 25% for DG region) could be observed in the NASH group in contrast to ND group (*p* < 0.05, two-way ANOVA and Tukey), nevertheless, compared with the NASH group, and an upward trend was noted in the PAMK group (10% for CA3, 12% for CA1 and *p* < 0.05, 5% for DG region) (Figure 4A). To further determine whether changes in hippocampal neurons were associated with the expression of proinflammatory factors, we performed RT-PCR to detect the mRNA expression levels of IL-1β, TNF-α, IL-6, and MCP-1 in the hippocampus of three groups. Figure 4B–E illustrated that compared with the ND group, proinflammatory factors including IL-6, IL-1β, TNF-α, and MCP-1 increased almost 2-fold in the NASH group, and PAMK intervention effectively reduced this increment (*p* < 0.05, one-way ANOVA and Dunnett’s post hoc test). Thus, PAMK might exert a protective action on the hippocampus by reducing damage and inflammation of neurons in NASH mice.

### 3.5. Prebiotics like PAMK Restoring the Structure and Composition of Gut Microbiota in NASH Mice

**Figure 5:**
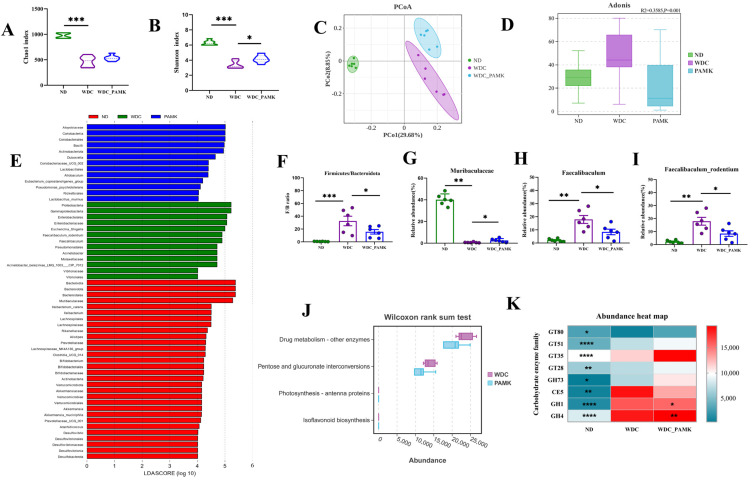
The effects of prebiotics-like PAMK on the gut microbiome in NASH mice. (**A**,**B**) The violin plot of Chao1 and Shannon index; *p*-value obtained by one-way ANOVA with Dunnett’s post hoc test. (**C**) Principal coordinate analysis (PCoA) based on unweighted unifrac. (**D**) Adonis analysis of similarities, *p* = 0.002 for ND vs. WDC group and *p* = 0.003 for WDC-vs.-PAMK group. (**E**) LAD score of 56 differential microbiota with LEfSe analysis in ND vs. WDC vs. PAMK group (LDA > 4, *p* < 0.05). (**F**) The ratio of Firmicutes to Bacteroidota. (**G**–**I**) Representative of the relative abundance of Muribaculaceae, Faecalibaculum and Faecalibaculum_rodentium. Data are presented as the mean ± SD (*n* = 6); *p*-value obtained by one-way ANOVA with Dunnett’s post hoc test. (**J**) Wilcoxon rank sum test of PICRUSt2 functional prediction based on level3 in WDC vs. PAMK group. (**K**) The heat map of relative abundance of gut microbiota obtained eight carbohydrate enzyme genes in carbohydrate metabolism pathway. * *p* < 0.05 or ** *p* < 0.01 or *** *p* < 0.001 or **** *p* < 0.0001 in ND vs. WDC or WDC vs. WDC_PAMK; *p*-value obtained by two-way ANOVA with Dunnett’s post hoc test.

Accumulating reports suggest that gut microbiota impacts the neurobiological features of depression and anxiety [41,42]. Particularly, dysbiosis of gut microbiota has emerged as a potential risk factor for NASH [43]. In this research, to find out the role of PAMK on gut microbiota, we performed 16S rRNA gene sequencing with mice fecal samples. While similarities in species richness were shown (Chao1 index), there was a significant difference in species evenness (*p* = 0.03 for Shannon index, one-way ANOVA and Dunnett’s post hoc test) between the NASH group and PAMK group (Figure 5A,B). The results of unweight unifrac principal component analysis (PCoA) and Adonis test illustrated that there were distinct differences between pairwise comparison groups (*p* = 0.002 for ND vs. WDC, *p* = 0.003 for WDC vs. PAMK, PerMANOVA for beta diversity) (Figure 5C,D). Additionally, we conducted LEfSe analysis on three groups with LDA > 4, *p* < 0.05, and 57 differential microbiotas were identified (Figure 5E). The ratio of Firmicutes to Bacteroidetes proved to rise in NAFLD [44], which also increased rapidly in the NASH group, and there was a significant decrease in the PAMK group (Figure 5F).

With relative abundance >0.1% and LDA >4 in both the ND vs. WDC and WDC vs. PAMK groups, we found the relative abundance of three microbiota (Muribaculaceae, Faecalibaculum, and Faecalibaculum_rodentium) was distinctly improved after PAMK intervention. Additionally, a previous study suggested that polysaccharides from Rosa roxburghii Tratt fruit could attenuate intestinal barrier dysfunction and inflammation in mice by increasing the abundance of Muribaculaceae [45]. In our research, we also observed that the relative abundance of Muribaculaceae expanded in the PAMK group (*p* = 0.016 for WDC vs. PAMK) (Figure 5G). Interestingly, compared to the ND group, NASH mice displayed a notable increase in relative abundance of Faecalibaculum_rodentium which belonged to Firmicutes phylum, while PAMK administration alleviated this effect, and a similar trend of change could be observed in Faecalibaculum (Figure 5H,I). It was worth noting that Muribaculaceae (LDA = 5.59, *p* < 0.001) and Faecalibaculum_rodentium (LDA = 5.24, *p =* 0.003) were the two genera with high contribution to the difference of the three groups. Furthermore, PICRUSt2 functional prediction suggested that the taxa of microbiota in the NASH group significantly enriched the Pentose and glucuronate interconversions pathway as compared to the PAMK group (Figure 5J). It is reported that carbohydrate enzymes can hydrolyze various dietary fibers and resistant starch to provide nutrients for the proliferation of gut microbiota [46]. To further clarify whether carbohydrate enzymes on the carbohydrate metabolism pathway changed, the abundance of genes in related enzymes were predicated, and we discovered that prebiotics like PAMK exerted an improvement effect on glycosyltransferases, carbohydrate esterase, and the glycoside hydrolases family, and significantly adjusted the abundance of glycoside hydrolases 1 (GH1) and glycoside hydrolases 4 (GH4) genes to near normal levels (Figure 5K). To summarize, prebiotics like PAMK adjusted gut microbial composition and structure in NASH mice which might be related to specific carbohydrate enzymes.

### 3.6. Serum Non-Targeted Metabolites Were Modulated by Prebiotics Like PAMK in NASH Mice

**Figure 6:**
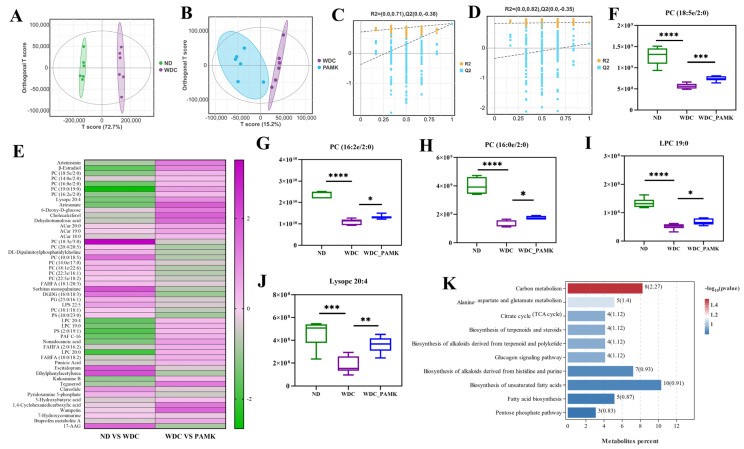
Prebiotics-like PAMK altered the serum metabolites in NASH mice. (**A**,**B**) OPLS-DA score chart of ND vs. WDC group and WDC vs. PAMK group. (**C**,**D**) Permutation test Chart of OPLS-DA score chart of pairwise comparisons. (**E**) The relationship between up- and down-regulation of 51 differential serum metabolites in ND vs. WDC group and WDC vs. PAMK group, and legend represents the multiple of up and down adjustment. (**F**–**J**) Representative of serum metabolites PC (18:5e/2:0), PC (16:2e/2:0), PC (16:0/2:0), Lysopc 20:4, LPC 19:0. Data are presented as the mean ± SD (*n* = 6), * *p* < 0.05 or ** *p* < 0.01 or *** *p* < 0.001 or **** *p* < 0.0001; *p*-value obtained by one-way ANOVA with Dunnett’s post hoc test. (**K**) *p*-value top 10 pathways of KEGG enrichment analysis. The numbers on the columns indicate the number of metabolites enriched in the pathway, and the number in brackets indicates *p*-value after taking −log10.

To further reveal metabolic phenotypes, we performed untargeted metabolic analysis on the serum from Normal, NASH, and PAMK mice. OPLS-DA showed that there were distinctive clusters in three groups (Figure 6A,B). The permutation test indicated that no overfitting phenomenon existed in the OPLS-DA model (Figure 6C,D). After quality control and normalization, we detected a total of 1212 serum metabolites. Particularly, there were 51 significantly different metabolites including lipids and lipid-like molecules, benzenoids, organ heterocyclic compounds, phenylpropanoids, and polyketides after PAMK intervention (Figure 6E). Moreover, to investigate the potential function of PAMK-related metabolites, with VIP > 1, *p* < 0.05, we obtained five common serum metabolites including PC (18:5e/2:0), PC (16:2e/2:0), PC (16:0/2:0), Lysopc 20:4, and LPC 19:0 of the ND, NASH, and PAMK groups (Figure 6F–J). Additionally, we found six long-chain fatty acids were enriched in model mice, and especially compared with the normal group, the concentrations of trans-10-heptadecenoic acid, tetradecane dioic acid, and docosatrienoic acid were significantly increased in the NASH group (Figure S1A). The concentrations of medium chain fatty acids decreased in NASH mice, but after intervention with PAMK, which tended to approach normal levels (Figure S1B). Subsequently, Kyoto Encyclopedia of Genes and Genomes (KEGG) enrichment analysis displayed that there was significant enrichment of serum metabolites in carbon metabolism, alanine, and aspartate and glutamate metabolism pathways (*p* < 0.05). There were still abundant metabolites modestly, but not significantly, enriched in the biosynthesis of unsaturated fatty acids, fatty acid biosynthesis pathway, Citrate cycle pathway, and pentose phosphate pathway (*p* > 0.05) (Figure 6K). In a nutshell, prebiotics like PAMK engendered advantageous effects on NASH by adjusting serum lipids and lipid-like molecules, which might be related to the carbon metabolism pathway, biosynthesis of unsaturated fatty acids, fatty acid biosynthesis pathway, and Citrate cycle pathway.

### 3.7. Integrated Correlation Analysis Elucidated the Relationship Between Microbiota, Serum Metabolites, Proinflammatory Factors, and Behavioral Indicators

**Figure 7:**
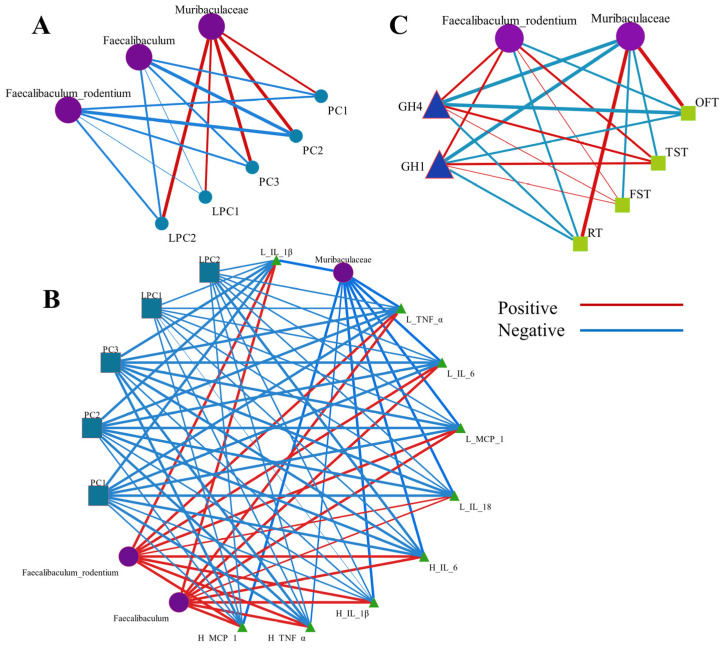
Correlation between gut microbiome and proinflammatory factors and serum metabolites. (**A**) The network diagram of positive and negative relationships between differential gut microbiota and serum metabolites; PC1~PC3: PC (18:5e/2:0), PC (16:0e/2:0), PC (16:2e/2:0); LPC1~LPC2: Lysopc 20:4, LPC 19:0. (**B**) The network diagram of positive and negative relationships between gut microbiota, serum metabolites and proinflammatory factors in liver and hippocampus; L: Liver; H: Hippocampus. (**C**) The correlation heatmap between specific gut microbiota and behavioral experimental parameters and GH1, GH4; OFT: time in center area of open field test; TST: immobility time in tail suspension test; FST: immobility time in forced swimming test; RT: latency time in rotarod test. The *r*-value in Spearman analysis was used to reflect the degree of correlation. Red color represents positive correlation and blue represents negative correlation, and the thickness of line represents the degree of correlation in network diagram.

To elucidate the interplay between gut microbiota and serum metabolites, and proinflammatory factors, a Spearman correlation analysis was conducted. We found PC (18:5e/2:0), PC (16:0e/2:0), PC (16:2e/2:0), and LPC 19:0 had a notable negative link with Faecalibaculum_rodentium. As the species-level microbiota of Faecalibaculum and proinflammatory factors in the liver and hippocampus (0.6 < /*r*/ < 0.8, *p* < 0.05), there was an extremely strong positive correlation between Muribaculaceae and PC (18:5e/2:0), PC (16:0e/2:0), PC (16:2e/2:0), and LPC 19:0 (*r* > 0.8, *p* < 0.05), and Muribaculaceae showed a substantially positive correlation with Lysopc 20:4 (*r* = 0.6, *p* < 0.05) (Figure 7A,B). Furthermore, Figure 7B displays that there was a moderately strong positive tie between Faecalibaculum_rodentium and several proinflammatory factors in the hippocampus (0.6 < *r* < 0.8, *p* < 0.05), and a marked positive correlation in the liver (*r* ≥ 0.8, *p* < 0.05). Different from Faecalibaculum_rodentium, Muribaculaceae was observed to have an opposite relationship with proinflammatory factors. Previous research demonstrated that Faecalibaculum_rodentium is closely associated with anhedonia depression-like behaviors in mice [47], and Muribaculaceae played an important role in alleviating anxiety-like behaviors in mice [48]. Hence, to deeply clarify the relationship between Faecalibaculum_rodentium, Muribaculaceae, and NASH-related anxiety/depression-like behaviors, we performed a Spearman correlation analysis. The results displayed that Faecalibaculum_rodentium was significantly positively associated with immobility time (*r* = 0.77 for TST, *r*= 0.58 for FST), and had a negative correlation with the time in the center in OFT (*r* = −0.67) as well as latency time in RT (*r* = −0.79) (Figure 7C). In contrast, Muribaculaceae showed a strong positive association with the time in the center area in OFT (*r* = 0.82) and latency time in RT (*r* = 0.85), accompanied by a significantly converse association with immobility time (*r* = −0.74 for TST, *r* = −0.64 for FST) (Figure 7C). Meanwhile, the abundance of GH1 and GH4 genes displayed a negative correlation with Muribaculaceae and a positive association with Faecalibaculum_rodentium and anxiety/depression-like behaviors. In general, our results demonstrated gut microbiota, particularly Muribaculaceae, Faecalibaculum_rodentium, and serum metabolites were deeply associated with NASH and related anxiety/depression-like behaviors.

## 4. Discussion

Accumulated research displayed that high-fat and high-cholesterol diets, irregular lifestyle, and gut microbiota imbalance are the main cause of NASH and systemic inflammation [49,50]. NASH is associated with mental burden and worsens as the disease progresses. Meanwhile, a meta-analysis showed that patients with nonalcoholic fatty liver disease have comorbid depression and anxiety [51,52]. The pathogenesis of NASH involves multiple mechanisms including lipid metabolism disorder, and alteration of the immune response, gut microbiome imbalance, and the disruption of the gut-liver axis and micro-derived metabolites are the main causes [6]. Additionally, the development from NAFL to NASH involves “multiple hits”, and the second hit includes oxidative stress in mitochondria and endoplasmic reticulum, which is the main reason for the progression of NASH to fibrosis [53]. Endotoxin of gut microbiota could also lead to systemic inflammation [54]. Coincidently, peripheral inflammation could induce depressed motivational behaviors [55]. Served as a prebiotic, plant polysaccharides could exert a potentially protective effect on human health via gut microbiome [56]. Polysaccharides of Atractylodes macrocephala Koidz (PAMK) from Shenling Baizhu San have the functions of preventing blood glucose and lipid metabolism disorders, liver protection, and anti-inflammatory properties [57]. However, the effect of PAMK on the liver and related behaviors is not fully understood. Thus, this study induced the NASH mouse model via a Western diet and microinjected CCl_4_ to reveal the therapeutic effects of PAMK on NASH and related anxiety/depression-like behaviors.

This study found PAMK effectively modulated liver lipids and functions, suppressed glucose intolerance, and reduced anxiety/depression-like behaviors in mice. The human liver is the main organ for synthesizing and storing cholesterol, and the increase in TC and TG might be related to fatty liver. Anxiety and depression, as NASH-related symptoms burden, seriously affect human quality of life [39]. In our results, PAMK decreased the concentrations of liver TC, TG, AST, and ALT and improved IPGTT, which was similar to a previous study [55]. In addition, PAMK also adjusted anxiety/depression-like behaviors in mice. Therefore, PAMK could act as a prevention for the development of NASH and related anxiety/depression-like behaviors in mice. Next, we explore the effect of PAMK on the pathological mechanism of NASH. Evidence suggested that the clinical pathological characteristics of NASH were steatosis, hepatocyte damage, and inflammatory infiltration, with or without fibrosis [58]. Systemic inflammation-induced damage or inflammation of neuronal cells in the hippocampus can lead to mood and mental disorder [59,60]. The study we performed found that PAMK could not only inhibit liver steatosis, improving fibrosis and inflammation, but also reduce neuroinflammation in mice. Other research also showed that an increase in the proinflammatory factors IL-1β and IL-18 was associated with the upregulation of the NLRP3 inflammasome complex in NASH mice [61]. In turn, these changes were responsible for chronic systemic inflammation [62,63,64], which were vital factors driving anxiety/depression-like behaviors [65]. Thus, PAMK inhibited inflammation both in the liver and hippocampus.

Multi-omics results displayed that the Western diet not only disrupted the structure and composition of gut microbiota, but also the balance of serum metabolism, which was consistent with the inflammation in the liver and hippocampus. Fortunately, PAMK intervention partly restored such disturbance. The ratio of Firmicutes/Bacteroidetes (F/B) at the phylum level was considered a risk factors for NAFLD [44], and the increase in the F/B ratio could be observed in both humans and mice following a high-fat diet. Other research has pointed out that serum lipid metabolites altered significantly in patients from normal to NASH [66]. In these current results, PAMK obviously reduced the F/B ratio and upregulated specific serum lipids molecules. It could be considered that PAMK improved the diversity and structure of gut microbiome, and regulated the imbalance of serum metabolism.

Belonging to the Erysipelotrichaceae family, Faecalibaculum_rodentium possessing carbohydrate metabolism-related gene clusters could achieve expansion in a high-fat, high-sugar diet environment, and reduced protective intestinal *Th17* cells as well as glucose tolerance in mice [67,68]. Apart from this, Faecalibaculum_rodentium was also considered to be deeply related to intestinal inflammation and liver steatosis [69]. Simultaneously, the occurrence of NASH is related to endoplasmic reticulum (ER) stress. ER is responsible for protein folding, while unfolded protein response (UPR) in ER induces liver cell inflammation, inflammasome activation, and death [70,71]. Concurrently, Faecalibaculum, which is in the same developmental branch of Faecalibaculum_rodentium, was highly relevant to serum IL-6, IL-1β, and TNF-α, and depressive-like behaviors [47]. Furthermore, the study confirmed that Ephx2 knockout (KO) mice do not exhibit depression-like behaviors even when exposed to chronic social defeat stress. Yet Ephx2(KO) mice transplanted with Faecalibaculum_rodentium reduced the expression of synaptic proteins in the prefrontal cortex through systemic inflammation, which results in anhedonia depressive-like behaviors, accompanied by an increase in IL-6 in serum [72]. Similarly, in this study, mice in the NASH group not only experienced a significant expansion of Faecalibaculum_rodentium, but also exhibited anxiety/depression-like behaviors, and inflammation also occurred in the liver and hippocampus. Fatefully, PAMK inhibited the expansion of Faecalibaculum_rodentium and alleviated anxiety/depression-like behaviors and neuroinflammation. Moreover, it is reported that the disturbances in lipid composition influenced the membrane fluidity and membrane proteins of ER, which promoted the activation of UPR [73]. Previous studies also indicated that low concentrations of plasma LPCs and PCs are linked to NASH [74], which is similar to our results. Additionally, medium-chain fatty acids, as a major anti-inflammatory ingredient in a beneficial diet, could suppress lipotoxicity-induced hepatic fibrosis in mice [75,76]. In our results, under the Western diet pattern, medium-chain fatty acid levels decreased, but there was a trend of improvement after PAMK intervention in mice. Therefore, the inhibitory effect of PAMK on Faecalibaculum_rodentium and the improvement of serum lipid metabolism suggested that it had a potential protective effect against inflammation in mice fed with a Western diet.

Further pathway analysis revealed that the abundance of microbiota possessing carbohydrate metabolism-related GH1 and GH4 genes in the NASH group was significantly enriched in the Pentose and glucuronate interconversions pathway as compared to PAMK group, and serum metabolites were enriched in the Carbon metabolism and Citrate cycle pathways. Existing literature indicates that the overaction of the aforementioned pathways was proven to be involved in obesity, insulin resistance, synthesis of inflammatory mediators, and Oxidative stress [77,78]. Simultaneously, other studies indicated that depression is associated with enrichment of glycoside hydrolases and polysaccharide cleavages [79], which is consistent with our results. Hence, we speculated that PAMK might adjust bidirectional effects between gut microbiota, serum metabolites, liver, and hippocampus by affecting the activity of carbohydrate enzymes, inhibiting inflammatory reactions, and improving glycolipid metabolism disorder.

There are still some limitations in this study. First, although mice and humans share 85% of common genes, overlap in overall intestinal layout and functions, and similarities in microbiota community composition at the phylum level, different body sizes, dietary habits, and metabolic rates are the main reasons for the translatability issues when transferring research results from rodent models to humans. Also, the reconstruction of PICRUST2′s functional contribution is bioinformatic and predictive. In addition, established NASH animal models can accurately represent some aspects of NASH. However, they cannot fully simulate human NASH pathology, which will affect our evaluation of the efficacy of PAMK on NASH.

## 5. Conclusions

In this study, NASH mice induced by Western diet not only exhibited liver steatosis, inflammation, fibrosis, and glucose tolerance disorder, but also exhibited anxiety/depression-like behaviors. More importantly, further multi-omics analysis revealed that PAMK as a potential prebiotic ameliorated NASH and associated anxiety/depression-like behaviors in mice, probably by regulating Faecalibaculum_rodentium, carbohydrate enzymes, and lipid metabolites. This will provide an important experimental basis for prebiotics like polysaccharides to improve NASH and reduce the mental burden associated with NASH. Therefore, future studies should use in vivo models and human samples to fully validate the efficacy and mechanisms of PAMK.

## Data Availability

The data that support the findings of this study are available from the corresponding author upon reasonable request.

## Supplementary Materials

The following supporting information can be downloaded at: https://www.mdpi.com/article/10.3390/nu16223837/s1, Figure S1: The concentrations of long and medium chain fatty acids; Table S1: The primer sequence of target gene used in the study; Table S2: Reagents used in the experiment.
