# Supplementary material for: PAMK Ameliorates Non-Alcoholic Steatohepatitis and Associated Anxiety/Depression-like Behaviors Through Restoring Gut Microbiota and Metabolites in Mice
Source: Nutrients. 2024 Nov 8;16(22):3837. doi: 10.3390/nu16223837 (PMC11597619; doi:10.3390/nu16223837)
Supplement: Supplementary file 1 [file nutrients-16-03837-s001.zip › nutrients-3282428-supplementary.pdf]

Supplemental Table S1. The primer sequence of target gene used in the study.

Gene	Primer sequences (5′- 3′)
**IL-6**	Forward: GTGACAACCACGGCCTTCCCTACT<br>Reverse: GGTAGCTATGGT ACTCCA
**TNF-α**	Forward: GCGACGTGGAACTGGCAGAAG<br>Reverse: GGTACAACCCATCGGCTGGCA
**IL-1β**	Forward: GAAATGCCACCTTTTGACAGTG<br>Reverse: TGGATGC TCTCATCAGGACAG
**MCP-1**	Forward: TCTG GGCCTGCTGTTCACA<br>Reverse: GGATCATCTTGCTGGTGAATGA
**IL-18**	Forward: ACAACTTTGGCCGACTTCAC<br>Reverse: ATCAGTCTGGTCTGGGGTTC
**GAPDH**	Forward: TCAACAGCAACTCCCACTCTTCCA<br>Reverse: TTGTCATTGAGAGCAATGCCAGCC

Supplemental Table S2. Reagents used in the experiment

**Reagent**	**Resource**	**Identifier**
Carbon tetrachloride (CCL_4)	Sinopharm Chemical Reagent Co., Ltd., China	Cat#: 10006464
Corn oil	Sigma	Cat#: 8001-30-7
Ethyl Alcohol	FuYu Chemical, China	Cat#: 64-17-
Dimethylbenzene	Guangzhou Chemical reagent factory, China	Cat#:1330-20-7
Haematoxylin staining solution	Biosharp, China	Cat#: BL702A
Eosin Y Solution	Solarbio, China	Cat#: G1100
Rhamsan gum	Sinopharm Chemical Reagent Co., Ltd., China	Cat#: 10004160
Glycerol Jelly Mounting Medium	Beyotime, China	Cat#: C0187
**Critical commercial assays**	**Resource**	**Identifier**
Serum alanine aminotransferase (ALT)	Nanjing Jiancheng Bioengineering Institute, China	Cat#: C009-2-1

Serum aspartate aminotransferase (AST)	Nanjing Jiancheng Bioengineering Institute, China	Cat#: C010-2-1
Hepatic triglyceride (TG)	Nanjing Jiancheng Bioengineering Institute, China	Cat#: A110-1-1
Hepatic total cholesterol (TC)	Nanjing Jiancheng Bioengineering Institute, China	Cat#: A111-1-1
RNA extraction kit	Vazyme, America	Cat#: RC101
DNA conversion kit	Vazyme, America	Cat#: R223-01
Quantitative PCR kit	Vazyme, America	Cat#: Q712
Glucose kit	Accu-Chek, Hong Kong	Cat#: L0140
Oil Red O Stain Kit	Nanjing Jiancheng Bioengineering Institute, China	Cat#: D027-1-1
Histochemistry kit DAB colorant	Wuhan Servicebio Technology CO., LTD, China	Cat#: G1212
Modified Sirius Red Stain Kit	Beijing Solarbio Technology CO., LTD. China	Cat#G1472

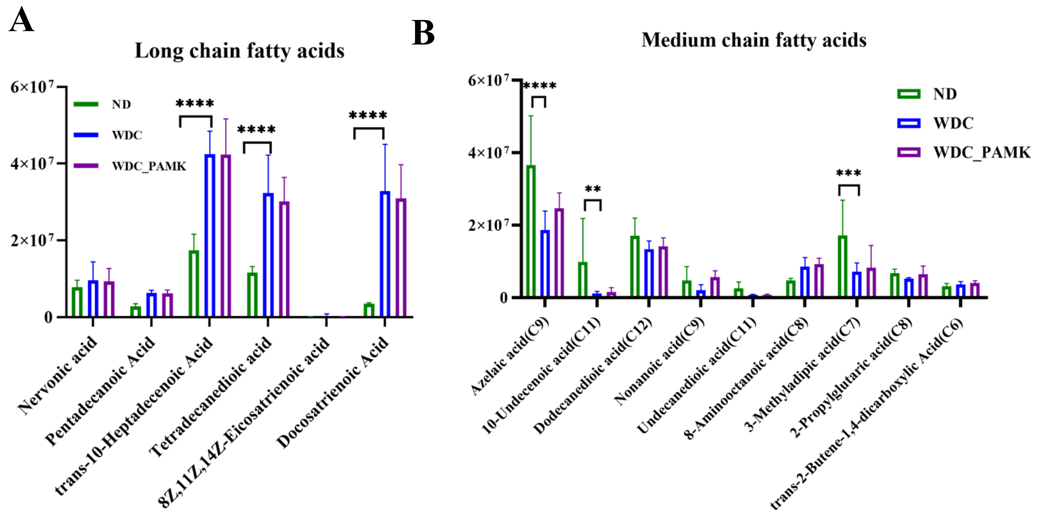

Supplemental Figure S1. (A) The concentrations of six long chain fatty acids. **(B)** The concentrations of nine medium chain fatty acids. Two-way ANOVA with Tukey post hoc test. Data are presented as the mean ± SD (n=6), * $P < 0.05$ or ** $P < 0.01$ or *** $P < 0.001$ or ****$P < 0.0001$.
